# Crosstalk Between Peroxisome Proliferator-Activated Receptor Gamma and the Canonical WNT/β-Catenin Pathway in Chronic Inflammation and Oxidative Stress During Carcinogenesis

**DOI:** 10.3389/fimmu.2018.00745

**Published:** 2018-04-13

**Authors:** Alexandre Vallée, Yves Lecarpentier

**Affiliations:** ^1^DRCI, Hôpital Foch, Suresnes, France; ^2^Centre de Recherche Clinique, Grand Hôpital de l’Est Francilien (GHEF), Meaux, France

**Keywords:** canonical WNT/β-catenin pathway, PPARγ, carcinogenesis, chronic inflammation, oxidative stress, reactive oxygen species, cancer

## Abstract

Inflammation and oxidative stress are common and co-substantial pathological processes accompanying, promoting, and even initiating numerous cancers. The canonical WNT/β-catenin pathway and peroxisome proliferator-activated receptor gamma (PPARγ) generally work in opposition. If one of them is upregulated, the other one is downregulated and *vice versa*. WNT/β-catenin signaling is upregulated in inflammatory processes and oxidative stress and in many cancers, although there are some exceptions for cancers. The opposite is observed with PPARγ, which is generally downregulated during inflammation and oxidative stress and in many cancers. This helps to explain in part the opposite and unidirectional profile of the canonical WNT/β-catenin signaling and PPARγ in these three frequent and morbid processes that potentiate each other and create a vicious circle. Many intracellular pathways commonly involved downstream will help maintain and amplify inflammation, oxidative stress, and cancer. Thus, many WNT/β-catenin target genes such as c-Myc, cyclin D1, and HIF-1α are involved in the development of cancers. Nuclear factor-kappaB (NFκB) can activate many inflammatory factors such as TNF-α, TGF-β, interleukin-6 (IL-6), IL-8, MMP, vascular endothelial growth factor, COX2, Bcl2, and inducible nitric oxide synthase. These factors are often associated with cancerous processes and may even promote them. Reactive oxygen species (ROS), generated by cellular alterations, stimulate the production of inflammatory factors such as NFκB, signal transducer and activator transcription, activator protein-1, and HIF-α. NFκB inhibits glycogen synthase kinase-3β (GSK-3β) and therefore activates the canonical WNT pathway. ROS activates the phosphatidylinositol 3 kinase/protein kinase B (PI3K/Akt) signaling in many cancers. PI3K/Akt also inhibits GSK-3β. Many gene mutations of the canonical WNT/β-catenin pathway giving rise to cancers have been reported (CTNNB1, AXIN, APC). Conversely, a significant reduction in the expression of PPARγ has been observed in many cancers. Moreover, PPARγ agonists promote cell cycle arrest, cell differentiation, and apoptosis and reduce inflammation, angiogenesis, oxidative stress, cell proliferation, invasion, and cell migration. All these complex and opposing interactions between the canonical WNT/β-catenin pathway and PPARγ appear to be fairly common in inflammation, oxidative stress, and cancers.

## Introduction

Cancer is a complex process that can be defined in term of three steps: initiation, promotion, and progression (1). Several chemical, physical, and biological factors may induce chronic inflammation, thereby increasing the risk of cancers (2). This link between cancer and inflammation has been reported in experimental and epidemiological studies (3, 4) and demonstrated through the efficacy of anti-inflammatory therapies in cancer (5). Chronic inflammation is responsible for various steps involved in carcinogenesis, such as promotion, survival, cellular transformation, invasion, proliferation, angiogenesis, and metastasis (6, 7).

Oxidative stress also operates at these stages by promoting DNA damages and genes mutations (8). In recent years, several studies have shown that the link between inflammation and cancer can involve oxidative stress through reactive oxygen species (ROS) production. Tumor promoters have the capacity to recruit inflammatory factors and then stimulate ROS production (9, 10). Oncogenic transformation is promoted by oxidative stress that acts as a DNA-damaging effector (11). ROS generation, together with oxidative stress, stimulates several signaling pathways that contribute to cancer development by regulating proliferation, invasion, angiogenesis, and metastasis (12).

The canonical WNT/β-catenin pathway regulates several signaling pathways involved in development and tissue homeostasis. This pathway is modulated from transcription level regulations to post-transcriptional modifications. An aberrant WNT/β-catenin pathway is observed in cancers (13, 14). This results in stimulating the expression of numerous WNT target genes involved in tumor development, such as c-Myc, cyclin D1, and HIF-1α (15), the production of ROS (16), and the activation of chronic inflammation (17).

In contrast, peroxisome proliferator-activated receptor gamma (PPARγ) is downregulated in numerous cancers (13). By regulating lipid and glucose homeostasis, differentiation, ROS and inflammation, PPARγ agonists appear to offer interesting therapeutic solution in cancers (18, 19).

In numerous tissues, canonical WNT/β-catenin pathway activation induces inactivation of PPARγ, while PPARγ activation induces inhibition of canonical WNT/β-catenin signaling (20). In most cancers, the canonical WNT/β-catenin pathway is increased while PPARγ is downregulated (13). PPARγ agonists induce repression of the canonical WNT/β-catenin signaling in several pathophysiological states. In this review, we focus on the crosstalk between canonical WNT/β-catenin pathway and PPARγ in chronic inflammation and oxidative stress during carcinogenesis processes.

## Peroxisome Proliferator-Activated Receptor Gamma

Peroxisome proliferator-activated receptors (PPARs) are ligand-activated transcription factors. Four subtypes of PPARγ have been identified: PPARγ1, PPARγ2, PPARγ3, and PPARγ4 (21, 22). After activation by natural or synthetic ligands, PPARγ heterodimerizes with the retinoid X receptor (RXR). Then, the complex PPARγ–RXR translocates to the nucleus to bind PPAR response elements (PPREs) to modulate the expression of several genes involved in immunity, inflammation, metabolism, cell proliferation, and cell differentiation (23–25). Fatty acids derivatives, such as 15-deoxy-delta-12,14-prostaglandin J (15d-PGJ2) hydroxyoctadecadienoic acid (9-HODE, 13-HODE), are endogenous ligands that activate PPARγ (26). PPARγ expression is involved in the development of heart and placenta (27) and during adipogenesis (28, 29). Thiazolidinediones (TZDs, antidiabetic drugs) are synthetic PPARγ ligands, which have been used in the diabetes treatment because of their ability to enhance insulin sensitivity. They also favor adipocyte differentiation and upregulation of adiponectin (30).

## Canonical WNT/β-Catenin Pathway

The WNT pathway is involved in numerous pathways that control tissue homeostasis and embryogenesis development. WNT ligands belong to the family of genes observed in humans, Xenopus, mice, drosophilia, and Zebrafish (31). Dysregulation of the canonical WNT pathway activity has been reported in several disorders and cancers (32–34).

**Figure 1:**
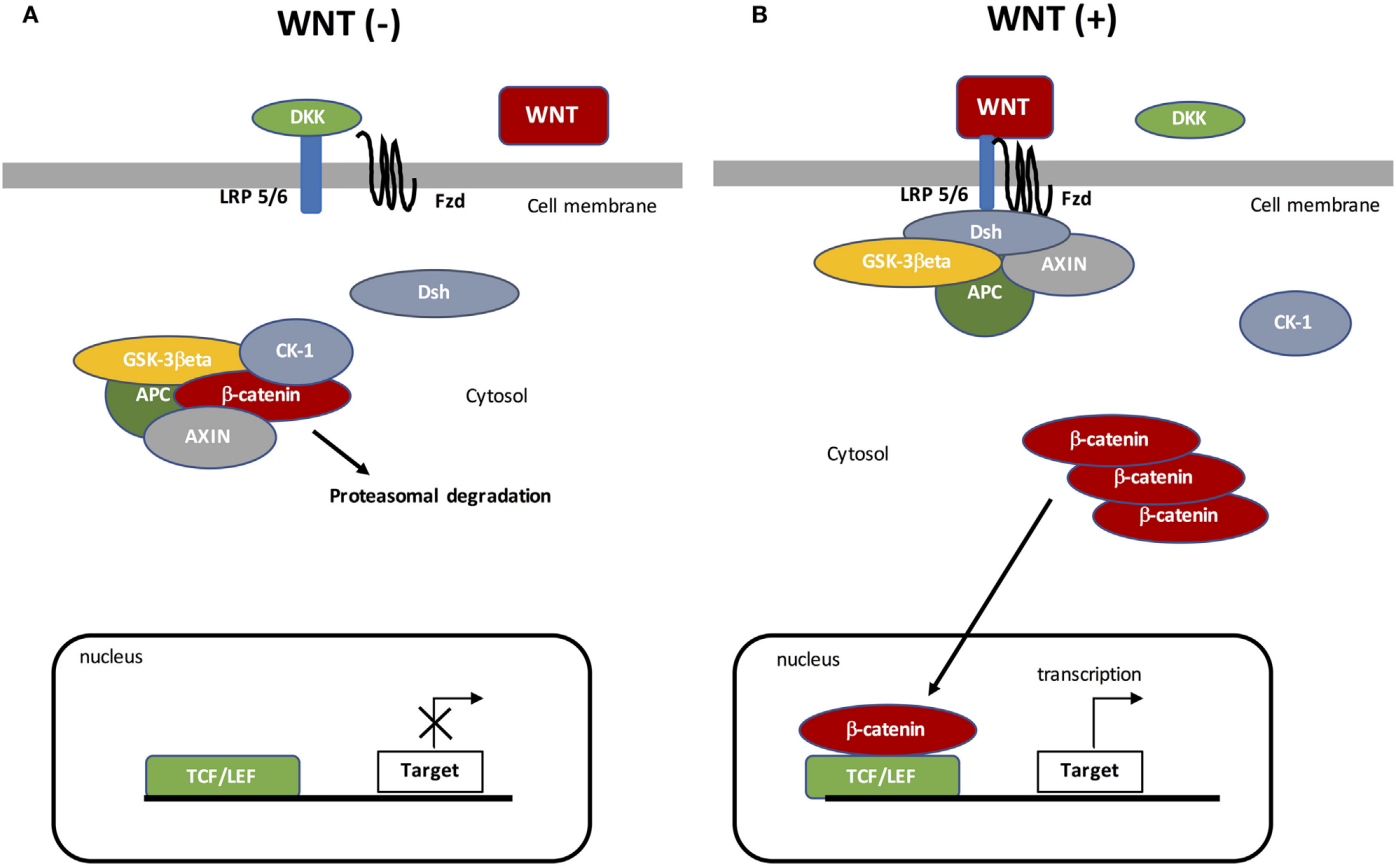
The canonical WNT/β-catenin pathway. **(A)** Under resting condition, the cytoplasmic β-catenin is bound to its destruction complex, consisting of adenomatous polyposis coli (APC), AXIN, and glycogen synthase kinase-3β (GSK-3β). After CK-1 phosphorylates on Ser45 residue, β-catenin is further phosphorylated on Thr41, Ser37, and Ser33 residues by GSK-3β. Then, phosphorylated β-catenin is degraded into the proteasome. Therefore, the cytosolic level of β-catenin is kept low in the absence of WNT ligands. If β-catenin is not present in the nucleus, the TCF/LEF complex cannot activate the target genes. DKK can inhibit the WNT/β-catenin pathway by binding to WNT ligands or LRP5/6. **(B)** When WNT ligands bind to both frizzled (FZD) and low-density lipoprotein receptor-related protein 5 (LRP5/6), Disheveled (DSH) is recruited and phosphorylated by FZD. Phosphorylated DSH in turn recruits AXIN, which dissociates the β-catenin destruction complex. Therefore, β-catenin escapes from phosphorylation and subsequently accumulates in the cytosol. The accumulated cytosolic β-catenin goes into the nucleus, where it binds to T-cell factor/lymphoid enhancer factor (TCF/LEF) and activates the transcription of target genes.

Canonical WNT signaling is characterized by the interaction between WNT ligand and specific targets resulting in cytosolic β-catenin accumulation and then its nuclear translocation (Figure 1). The nuclear activation of β-catenin results in the stimulation of downstream factors (35). During the “off state” of the WNT/β-catenin pathway, WNT ligands do not bind specific receptors. Cytosolic β-catenin is maintained at a minimal level through the activation of the β-catenin destruction complex, formed by the combination of AXIN (a cytoplasmic protein regulating G-protein signaling), glycogen synthase kinase-3β (GSK-3β, a serine-theronine kinase), adenomatous polyposis coli (APC, a tumor suppressor gene), and casein kinase 1 (CK-1, a serine/threonine-selective enzyme) (36). CK-1 and GSK-3β target β-catenin by phosphorylating the serine and threonine residues located in the amino acid terminus (37–39). CK-1 phosphorylates an N-terminus of β-catenin and GSK-3β phosphorylates a threonine 41 (Th41), Ser33, and Ser37 sites of β-catenin (35, 40). These phosphorylations result in recruiting APC in the destruction complex. APC modulates the degradation of the cytosolic β-catenin into the proteasome through its tumor suppressor properties (36, 41).

The “on state” is characterized by modified WNT/β-catenin signaling. WNT ligands bind Frizzled (FZD, a family of G protein-coupled receptors) and low-density lipoprotein receptor-related protein 5 (LRP 5/6) co-receptors (42). Then, Disheveled (DSH, a phosphoprotein) forms a complex with FZD, which results in the phosphorylation of LRP5/6 by GSK-3β and then the recruitment of the β-catenin destruction complex. DSH phosphorylates LRP6 (43), which inhibits GSK-3β activity leading to the stabilization and then the cytosolic accumulation of β-catenin. Accumulation of β-catenin leads to its nuclear translocation and then β-catenin binds the T-cell factor/lymphoid enhancer factor (TCF/LEF) transcription factors (44, 45). The nuclear complex formed by β-catenin and TCF/LEF activates several WNT target genes, such as c-Myc (a transcription factor) and cyclin D1 (a protein belonging to the highly conserved cyclin family encoded by the CCND1 gene) (37, 38). The WNT target genes are involved in several processes, such as cell division, proliferation, invasion, and stem cell maintenance (46). Furthermore, β-catenin accumulation is involved in cancer phenotype maintenance (47–49).

## Crosstalk between PPARγ and the WNT/β-Catenin Pathway

In several diseases, the WNT/β-catenin pathway and PPARγ act in an opposite manner as in cancers, such as gliomas (15, 50), neurodegenerative diseases, such as Alzheimer’s disease (51, 52), amyotrophic lateral sclerosis (53, 54), multiple sclerosis (55), age-related macular degeneration (56, 57), and fibrosis processes (58–60).

The WNT/β-catenin pathway and PPARγ interact through a TCF/LEF β-catenin domain and a catenin-binding domain within PPARγ (61–64). Downregulation of the WNT/β-catenin pathway leads to the stimulation of PPARγ expression (65), whereas PPARγ upregulation downregulates β-catenin levels in several cellular systems (66–68). PPARγ agonists stimulate synaptic plasticity by interacting with the WNT/β-catenin/phosphatidylinositol 3 kinase/protein kinase B (PI3K/Akt) pathway (69). Moreover, mesenchymal stem cell differentiation also presents this interaction between these two pathways (70).

Indeed, in numerous diseases, β-catenin signaling decreases PPARγ expression (71–80). In many studies, PPARγ operates as a negative β-catenin target gene (81, 82).

Peroxisome proliferator-activated receptor gamma agonists are considered as a promising treatment through the action of this crosstalk (83). Troglitazones (anti-inflammatory drugs) can decrease c-Myc levels (84). Intestinal fibrosis presents an activation of the WNT/β-catenin pathway, and the use of PPARγ agonists can decrease it and diminish fibrosis formation (85). PPARγ agonists activate Dickkopf-1 (DKK1, a WNT inhibitor) activity to decrease the canonical WNT/β-catenin pathway and then inhibit the fibroblasts differentiation (86). In 3T3-L1 cells, the inhibition of the signal transducer protein kinase B (Akt) pathway leads to activation of PPARγ (87). The phosphatidylinositol 3 kinase/protein kinase B (PI3K/Akt) pathway acts by phosphorylating GSK-3β to negatively regulate PPARγ expression (88, 89). Furthermore, PPARγ agonists activate GSK-3β to decrease β-catenin expression (90). Conversely, the β-catenin signaling activates the Akt pathway and this leads to a decrease in PPARγ expression in adipocytes and 2T2-L1 preadipocytes (68, 91). PPARγ agonists downregulate the PI3K/Akt signaling pathway (92, 93) by stimulating PTEN activity in fibrotic process (59).

Numerous inflammatory cytokines, chemokines, or intracellular pathways, such as the canonical WNT/β-catenin signaling, TNF-α, interleukin (IL)-1, and IL-13, downregulate PPARγ expression (94–96). The transcription factor COUP II is a canonical WNT target and downregulates PPARγ expression (97). In adipocytes, adiponectin increases PPARγ expression and then downregulates the LPS-induced NFκB expression and IL-6 production (98). Mesenchymal stem cell differentiation also shows a crosstalk between the WNT pathway and PPARγ (70). Hepatic fatty acid metabolism, fatty acid oxidation, hepatic mitochondrial function, and energy balance are regulated by the interaction between the WNT/β-catenin pathway and PPARγ (62, 99, 100).

### Crosstalk Between PPARγ and WNT/β-Catenin Signaling in Cancers

Even if, the molecular mechanisms by which TZDs regulate differentiation and stemness programs have been well studied in adipocytes and normal cells, in cancer cells, they still remain unclear (32). In normal cells, PPARγ suppresses tumorigenesis and WNT signaling by targeting phosphorylated β-catenin at the proteasome by a process involving its catenin-binding domain within PPARγ. In contrast, oncogenic β-catenin resists proteasomal degradation by inhibiting PPARγ activity, which requires its TCF/LEF-binding domain (62). In adipocytes, PPARγ increases differentiation and inhibits proliferation by affecting the WNT/β-catenin pathway. PPARγ interacts with GSK3-β to induce the differentiation factor C/EBPα and this leads to the production of adiponectin (101, 102). PPARγ activation reduces β-catenin at both the mRNA and protein levels to promote differentiation (103). In human metastatic prostate cancer LnCaP cells, PPARγ inhibits the WNT pathway by targeting phosphorylated β-catenin at the proteasome (62, 104). In gastric and colon cancer cells, PPARγ decreases β-catenin expression, subcellular localization, and downstream effectors, resulting in the modulation of several genes, such as telomerase reverse transcriptase, and Sox9, which are involved in cell development, differentiation, and survival processes (105–107). PPARγ agonists, by inhibiting activation of the WNT/β-catenin pathway, could be used in combination with other drugs such as inhibitors of tyrosine kinases (108), PI3K/AKT (109), and mitogen-activated protein kinase (MAPK) cascades to maximize the antitumor and pro-differentiating effect.

## Carcinogenesis: Role of Chronic Inflammation and Oxidative Stress

Cancer progression is promoted by an environment rich in inflammatory factors, DNA damages, and genetic or epigenetic mutations (110).

### Chronic Inflammation

Several studies have shown that prolonged inflammation leads to DNA damages and tissue injury (111). Chronic inflammation can affect cell homeostasis, metabolism, and genomic regulation, leading to the initiation of tumorigenesis (112). Furthermore, damages induced by chronic inflammation are responsible for the development of malignancy sites (113, 114).

The link between inflammation and cancer initiation has been examined in a recent study (115). Inflammation stimulates the activation of cytotoxic mediators, such as reactive oxygen species (ROS) and reactive nitrogen species (RNS), which have a major role in DNA damages (116). DNA damage accumulation is responsible for the initiation of carcinogenesis through the enhancement of genomic instabilities (117).

Pathogenic stimuli can stimulate inflammation and then eradicate the normal host defense response (118). Pathogens promote carcinogenesis through the recruitment of infections and the inhibition of immune response leading to chronic inflammation (3). Stomach, intestine, liver, colon, and skin are the main sites of common pathogenic infections that are believed to be related to cancer progression (119, 120).

The inflammatory response is regulated by the canonical WNT/β-catenin pathway (111). Moreover, infection pathogens can overexpress the WNT/β-catenin pathway leading to uncontrolled inflammation and then to an increased risk of carcinogenesis (121).

Several inflammatory factors can facilitate the migration and invasion of neoplastic cells (122). Tumor necrosis factor α (TNF-α), interleukin-6 (IL-6), vascular endothelial growth factor (VEGF), and tumor growth factor-β (TGF-β) are inflammatory factors involved in the regulation of the immune system (123). TGF-β and VEGF can suppress the immune response during cancer development (124). TNF-α overexpression induces DNA damage leading to tumor growth (122), angiogenesis, and invasion (125). TNF-α can stimulate other cytokines such as IL-17 to directly promote tumor growth (126). Then, IL-17 activates IL-6 and the signal transducer and activator transcription (STAT) signaling for invasion (127).

In parallel, chronic inflammation stimulates the expression of cyclooxygenase 2 (COX2, a prostaglandin-endoperoxidase synthase) (128). Numerous cytokines (TNF-α, IL-1) induce the activation of COX2 (122). COX2 is involved in the stimulation of ROS, and RNS intermediates found to be overexpressed during carcinogenesis processes (128, 129) and the production of prostaglandins leading to angiogenesis, anti-apoptosis, and metastasis (128, 130). Nuclear factor-kappaB (NFκB) activates numerous pro-inflammatory factors that induce COX2 and inducible nitric oxide synthase (iNOS) (112). NFκB is one of the main factors involved in inflammation in association with carcinogenesis (112, 131). NFκB activates the expression of TNF-α, IL-6, IL-8, COX2, BCL-2 (B-cell lymphoma 2), metalloproteinases (MMPs), and angiogenic factors such as VEGF (112), and ROS production (132).

The STAT3 pathway involved in metastasis, proliferation, and angiogenesis (133) is activated by VEGF and cytokines (IL-6). The STAT3 signaling appears over-activated in numerous cancers, such as colon, breast, stomach, prostate, skin, and head cancers (134). Moreover, iNOS, an enzyme which catalyzes nitric oxide (NO), is overexpressed during inflammation (135) and enhances p53 gene mutations (122).

### Oxidative Stress

Oxidative stress is characterized by an imbalance between production and elimination of reactive metabolites and free radicals (ROS and RNS) (8, 136). ROS generation is caused by cell damages through nitration and oxidation of macromolecules, such as proteins, lipids, DNA, and RNA. The NADPH oxidase (NOX) enzyme enhances ROS through the oxidation of intracellular NADPH to NADP^+^. Then, the transfer of electrons through the mitochondrial membrane reduces molecular oxygen and produces the superoxide anion as a primary product. ROS production has a major role in several pathways and in changes of intracellular and extracellular environmental conditions (137).

Reactive oxygen species are produced by dysregulation of the mitochondrial respiratory chain (138). During carcinogenesis, in a positive feedback, DNA damage and genomic instability can favor ROS production (139). ROS production has been observed in several cancer cells, such as in case of brain (140), breast (141), rachis (142), stomach (143), liver (144), lung (145), skin (146), pancreas (147), and prostate (148) cancers.

Leukocytes during inflammation are recruited from the damage sites and this leads to an increased uptake of oxygen, which induces the release of ROS and subsequently its accumulation (6, 149).

Several redox-regulated transcription factors have a key role in the stimulation of pro-inflammatory mediators, such as NFκB, a signal transducer and activator of transcription (STAT), activator protein-1 (AP-1), and the hypoxia-inducible factors (HIF) (112). The oxidative stress-induced inflammation induces the production of COX2, iNOS, TNF-α, IL-6, and miRNAs (150). A vicious circle operates between inflammation and oxidative stress leading to carcinogenesis (129).

NADPH-oxidase (NOX) is stimulated by inflammation and leads to oxidative stress and alteration of nuclear signaling (151). ROS, activated by NOX, stimulate the canonical WNT/β-catenin pathway through oxidization and inactivation of the nucleoredoxin (a redox-sensitive regulator), resulting in tumor cell proliferation (112). ROS production leads to the activation of c-Myc (152), STAT (153), and PI3K/Akt (154) and the inactivation of PPARγ (155). ROS production activates Akt signaling through the inhibition of the phosphatase and tensin homolog deleted from chromosome (PTEN) (156, 157). The Akt pathway is involved in cellular metabolism and the promotion of cell survival (156, 157).

## Interactions between the Canonical WNT/β-Catenin Pathway and Carcinogenesis

### Interactions Between the Canonical WNT/β-Catenin Pathway and Inflammation

**Figure 2:**
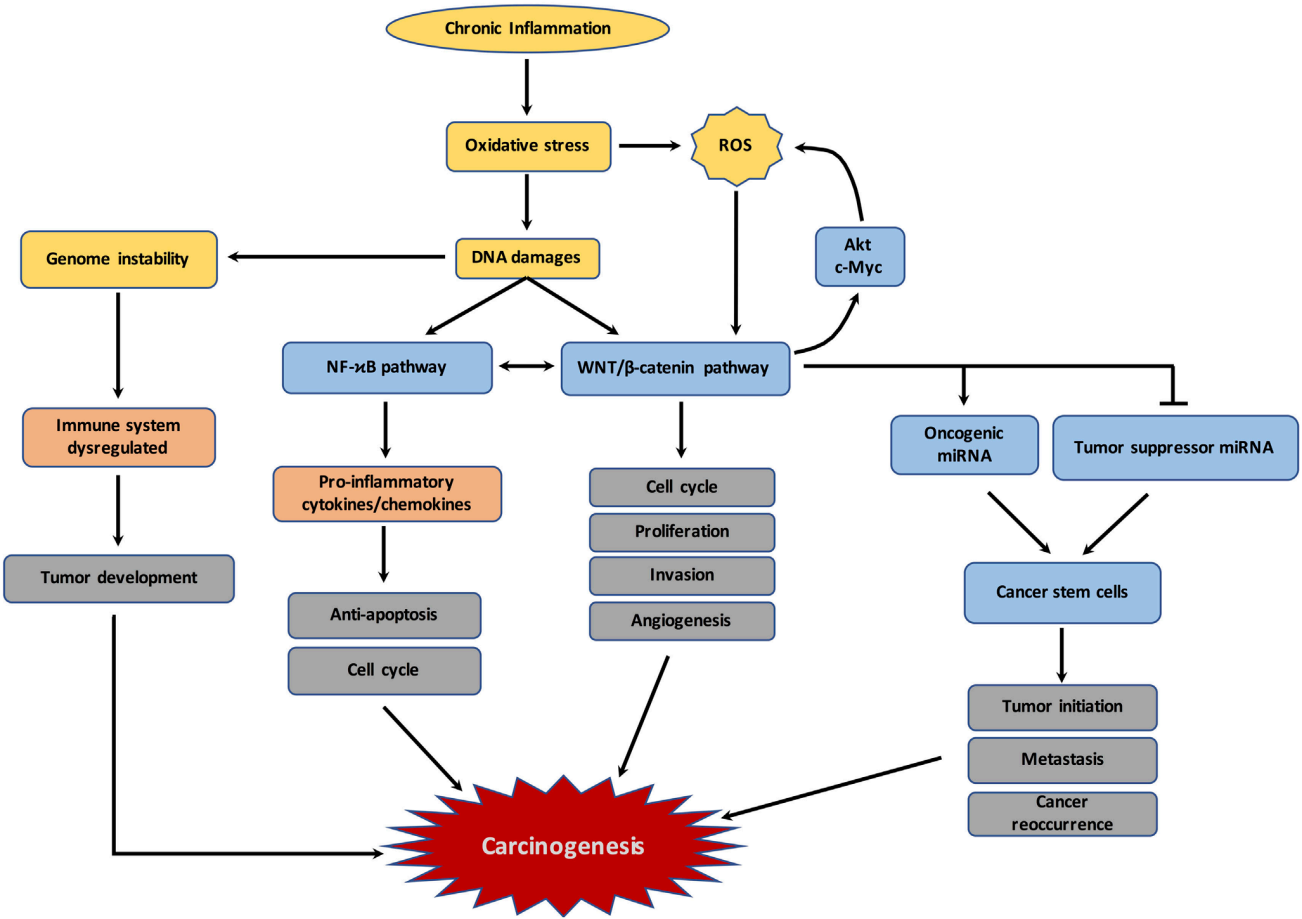
Schematic representation of the links between chronic inflammation, oxidative stress, DNA damages and carcinogenesis. The figure represents the different mechanisms involved by chronic inflammation and resulting in dysregulation of immune response, reactive oxygen species (ROS) production, DNA damages, and subsequently the initiation of carcinogenesis.

A positive crosstalk between WNT/β-catenin and NFκB has been reported recently (17). The overexpression of WNT/β-catenin results in the enhancement of IκB-α degradation and then NFκB transactivation (158) (Figure 2). Upregulation of the target gene, CRDBP, by stimulated β-catenin signaling leads to a stabilization of βTrCP mRNA (159). In colon cancer, overexpression of both βTrCP and CRD-BP is associated with the activation of the β-catenin signaling and NFκB, contributing to cell proliferation and metastasis (159, 160). In breast cancer, TLR3 stimulation activates β-catenin signaling simultaneously with activation of the NFκB pathway, in a synergistic manner (161). β-catenin and NFκB pathways act in together diffuse large B-cell lymphomas (162). The WNT/β-catenin pathway leads to an increase in COX expression, which then influences the inflammatory response (163). E-cadherin and GSK-3β are decreased in melanoma cells by the stimulated β-catenin signaling (164). Concomitant GSK-3β and E-cadherin inactivation with cytosolic β-catenin accumulation induces NFκB-dependent iNOS expression in hepatic cells (165). The WNT/β-catenin pathway modulates in a positive manner its downstream target TNFRSF19 in colon cancer, which activates the NFκB signaling (166). However, the synergistic effect between β-catenin and NFκB depends on both the TCF/LEF link and the context of the genes or cell types (167).

Nuclear factor-kappaB inhibits GSK-3β and positively regulates the β-catenin signaling (168, 169). Indeed, the activation of GSK-3β results in the inhibition of TNF-α-induced NFκB stimulation in carcinoma cells (168). IκB is stabilized by GSK-3β overexpression, which results in the inhibition of the NFκB pathway (169).

Nuclear factor-kappaB signaling can regulate WNT/β-catenin signaling through the use of IKKα (170) and RelA (171). IKKα can increase β-catenin signaling, whereas IKKβ downregulates β-catenin signaling (172). IKKα upregulates β-catenin/TCF/LEF activation and then the target gene cyclin D1 (173). GSK3-β and APC are degraded through the activation of IKKα leading to the cytosolic β-catenin accumulation (170). In glioma cells, overexpression of RelA coupled with the knockout of SN50, a NFκB inhibitor, increases the β-catenin nuclear translocation and then enhances β-catenin/TCF/LEF activity (174). The positive crosstalk between the WNT/β-catenin pathway and NFκB pathway participates in the regulation of several pathways involved in cancer development based on inflammation-induced carcinogenesis. This could be explained in part by the synergistic effect observed between β-catenin/TCF4 and NFκB on the overexpression of WNT target genes in colon cancer (171). This positive crosstalk induces several stem cell signature genes, such as Sox9, Ascl2, and Lgr5, leading to tumor growth.

### Interactions Between the Canonical WNT/β-Catenin Pathway and ROS

Reactive oxygen species production activates the PI3K/Akt pathway, which is overactivated in numerous cancers (175). PTEN is a phosphoinositide-3-phosphatase, which downregulates the PI3K/Akt pathway (157). NADPH oxidase and superoxide dismutase oxidize PTEN and inactivate it. Then, the inhibition of PTEN activity by oxidative stress increases the activity of Akt and thus enhances the phosphorylation of GSK-3β by Akt. GSK-3β inactivated by Akt does not inhibit the nuclear β-catenin signaling, resulting in cell proliferation in several cancers. Alkylation of PTEN activates Akt and β-catenin (176). In addition, ROS causes the stabilization of HIF-1α and then the activation of the glycolytic enzymes participating in cell proliferation and angiogenesis (50, 175). The WNT/β-catenin pathway can activate HIF-1α by stimulating the PI3K/Akt pathway (15).

A recent study by Zhang et al. (177) has shown that ROS production activates the WNT/β-catenin pathway, but the mechanism involved remains unclear (177) (Figure 2). Furthermore, carcinogenesis of cells can increase the endogenous level of ROS production (175). Indeed, several oncogenes enhance ROS production, such as Akt (16) and c-Myc (178).

### Genetic and Epigenetic Regulation of the Canonical WNT/β-Catenin Pathway in Cancers

Several genetic mutations lead to the aberrant activation of the canonical WNT/β-catenin pathway (14). In numerous malignant processes, the regulator genes of CTNNB1, AXIN, and APC have been observed to be mutated (179).

Mutations of CTNNB1, a β-catenin target, have been shown to be involved in the initiation of colon, gastric, ovarian, pancreatic, and prostate cancers, but also in melanoma and medulloblastoma (180, 181). APC mutations have been observed in colon cancer and AXIN mutations in hepatocellular carcinoma and medulloblastoma (182, 183).

Several studies have shown an interaction between miRNAs and the canonical WNT/β-catenin pathway, such as in osteoblast differentiation and cardiac and bone formation (184–186) (Figure 2). An aberrant expression of miR-374a is coupled with cytosolic β-catenin accumulation in breast cancers (187), and degradation of APC leading to the inactivation of the β-catenin destruction complex and enhancing the transcriptional activity of TCF/LEF (188). Moreover, the canonical WNT/β-catenin pathway controls the activity of several cancer-stem cell (CSC)-specific miRNAs. These specific miRNAs play a major role in tumor initiation. Overexpression of the WNT/β-catenin pathway leads to activation of the oncogenic miRNA expression to enhance the self-renewal potential of CSCs, which is involved in the resistance to drug therapy and initiation of new tumor growth (14).

Cancer-stem cells theory is characterized by the fact that cancer cells are derived from certain populations of cells, which possess stem cell properties (189–191). Several studies have shown that the WNT/β-catenin pathway can regulate stem cells and stem progenitors, plethora system maintenance, and cell self-renewal (37). Moreover, in a recent study, it was observed that the canonical WNT/β-catenin pathway plays a major role in the regulation of the activity of stem self-renewal in numerous cells (192).

The role of miRNAs in the regulation of CSCs is currently being investigated. Nevertheless, it has been observed that several miRNAs, such as miR410, can promote tumor growth, invasion, and migration of NSCLC cells through the activation of the canonical WNT/β-catenin pathway (193). miR-451 expression stimulates the upregulation of the macrophage migration inhibitory factor (MIF) and COX-2 expression to activate WNT/β-catenin pathway in CSCs (194, 195).

The interactions observed between WNT/β-catenin and miRNAs are involved in the regulation of tumorigenesis in numerous cancers, such as liver cancer (196), colon cancer (197, 198), brain cancer (199), and several other cancers (200–204).

On the other hand, other miRNAs appear to be tumor suppressors, such as miR-34a. miR-34a directly targets the tumor suppressor p53 and suppresses the expression of several target genes, such as SOX, Nanog, and N-Myc (205, 206). Let-7 is considered as a β-catenin negative regulator (207). Future studies will help us to better understand the role of miRNAs in the inhibition or activation of cancer initiation and its development through interaction with the WNT/β-catenin pathway by regulating the epithelial mesenchymal transition in cancer (208, 209).

## Action of PPARγ Agonists in Cancers

**Figure 3:**
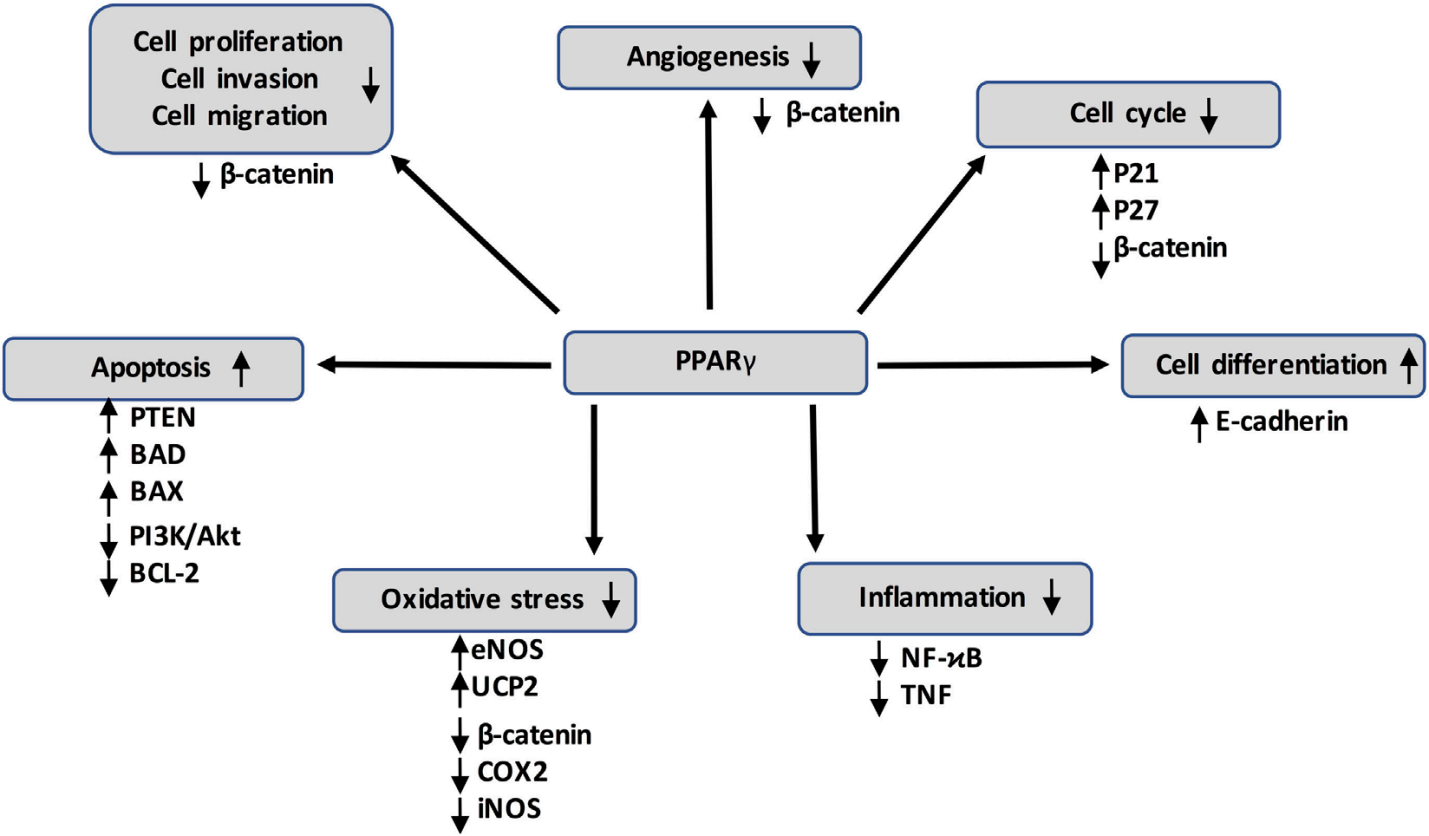
Peroxisome proliferator-activated receptor gamma (PPARγ) activation can inhibit cancer development through several mechanisms by acting on numerous target genes and pathways, such as the canonical WNT/β-catenin pathway. It also interferes with pro-inflammatory signaling by repressing nuclear factor-kappaB (NFκB) and tumor necrosis factor α (TNF-α).

Several studies have shown a significant reduction of PPARγ expression in cancers such as colon cancer (210, 211), gastric cancers (212), follicular thyroid cancer (213), cervical carcinoma (214), and esophageal cancer (215) (Figure 3). Numerous studies have shown that PPARγ has antineoplastic actions on lung, breast, prostate, and colon cancers (216, 217). NCOR, a repressor of PPARγ, has been found to increase in prostate cancer and to inhibit the expression of PPARγ (218). Several mutations of PPARγ are correlated with cancer initiation (219, 220). PPARγ agonists, such as rosiglitazone and troglitazone, are involved in cell cycle arrest, differentiation, proliferation, invasion, migration, apoptosis, inflammation, angiogenesis, and oxidative stress (19) (Table 1).

**Table 1:** Anti-tumoral effects of peroxisome proliferator-activated receptor gamma (PPARγ) agonists.

Effect	PPARγ agonists	Target	Cell line	Reference
Cell-cycle arrest	Troglitazone	p38 (activation)	Renal cell carcinoma	(1)
		Mitogen-activated protein kinase (MAPK) (inhibition)		
	Thiazolidinediones	p21 (activation)	Pancreatic cancer cells, human hepatoma cell lines	(221, 222)
	Troglitazone, Ciglitazone	p27 (activation)	Human hepatoma cell lines, pancreatic carcinoma cells	(222–225)
	Thiazolidinediones	β-catenin, cyclin D, estrogen receptor- alpha, IkappaB kinase (inhibition)	Breast cancer (MCF-1 and MCF-7 cell lines)	(226–230)
	Troglitazone, Ciglitazone, Rosiglitazone	Phosphatidylinositol 3 kinase/Protein kinase B (PI3K/Akt), ERK 1/2, MAPK (inhibition)	Prostate carcinoma (PC-3 cells)	(231)

Differentiation	Rosiglitazone	β-catenin (inhibition), SOX9 (inhibition)	Colon cancer (Caco2, SW480, HCT116, HT29 cells)	(106)
	pEGFP-N1-PPAR gamma recombinant plasmid	β-catenin (inhibition), SOX9 (inhibition)	Gastric cancer	(107)
	Thiazolidinediones	CEA, E-cadherin, p21 (activation)	Human pancreatic cancer cells (Capan-1, AsPC-1, BxPC-3, PANC-1, MIA PaCa-2 cells)	(221)
	Troglitazone	Ki-67 (inhibition)	Human liposarcoma	(232)
	Glitazone	p18, p21 (activation)	Pancreatic tumor cells, hepatocellular carcinoma cells	(216, 217)

Proliferation	Rosiglitazone	β-catenin (inhibition), glycogen synthase kinase-3β (GSK-3β) (activation)	Lymphoma cells	(104)
	pEGFP-N1-PPAR gamma recombinant plasmid	β-catenin (inhibition)	Gastric cancer (MKN)28, SGC-7901, BGC-823 cells	(105)
	Thiazolidinediones	Leptin receptor, cyclin D1 (inhibition)	Breast cancers	(233)
	Polyunsaturated fatty acids (PUFA)	Activator protein-1 (AP-1) (inhibition)	Human lung cancer (A549 cell lines)	(234)
	Omega-3 fatty acids	LDL, albumin (inhibition)	MCF-7 and PC-3 cells	(235)
	PUFA	Syndecan-1 (SDC-1) (activation)	Breast cancer (MCF-7 cell lines)	(236)
	Troglitazone	PSA production, sex hormone-binding globulin (SHBG) (inhibition)	Prostatic cancer (DU145 cells)	(231)
	Thiazolidinediones	Depletion of cytosolic Ca2^+^	Colon cancer	(237)
	Rosiglitazone	eIF2 (inhibition)	Liposarcoma	(237)
	Rosiglitazone	Cyclin D1 (inhibition)	NIH 3T3 and 3T3 L1 cells	(238)
	Pioglitazone	Ki-67, MMP-9 (inhibition), c-Jun N terminal protein kinase (activation)	Glioma cells (C6 cells)	(239)
	Troglitazone, Rosiglitazone, Prostaglandin J2 GW0233	VEGF (inhibition)	Prostate carcinoma (LNCaP, DU145, PC3 cells)	(240)

Apoptosis	Troglitazone	Bcl2 (inhibition), Bax (activation)	Gastric carcinoma cells (SGC790 cells)	(241)
	Rosiglitazone	PTEN (activation)	Human hepatocarcinoma (BEL-7404 cell line)	(242)
	Rosiglitazone, Lovastatin	PTEN (activation)	Breast cancer	(243)
	Rosiglitazone	PTEN (activation)	Human macrophages, Caco_2_ colorectal cancer cells, and MCF7 breast cancer cells	(244)
	Rosiglitazone	PTEN (activation)	Non-small cell lung cancer (A549 cells)	(245)
	Ciglitazone and Troglitazone	TNF-α/TRAIL (inhibition)	Human prostate cancer, PPC-1 and LNCaP, ovarian cancer, OVCAR-3, and SK-OV-3 cells	(246, 247)
	Troglitazone	Bcl-xl, Bcl2 (inhibition)	Prostate cancer cells (PC-3 cells)	(248)
	Rosiglitazone and KR-62980	PI3K/Akt (inhibition)	Breast cancer (MCF-7 cells)	(249)
	Troglitazone	Cdk2, E2F-1, cyclin B1, cyclin D3, PI3K (inhibition), p77 (activation)	Lung cancer (CL1-0, A549 cells)	(250)
	Troglitazone	Cyclin D1(inhibition)	Breast cancers (MCF-7, BT474, T47D, MDA-MB-231 cells)	(251)

Inflammation	Thiazolidinediones	NFκB, STAT3, TNF-α, IL-17, IL-6, Bcl2L11, CPNE7, FAS, HIF-1alpha, IL-1RAP, SOD2 (inhibition)	Colorectal, liver, bladder, lung, gastric neoplasm	(252–256)
	15d-PGJ(2)	NFκB (inhibition)	RAW264.7 cells	(257)

Oxidative stress	Rosiglitazone	NAD(P)H oxidase-derived superoxide (inhibition)	Coronary arterioles	(258)
	Troglitazone	Cu2+, Zn2+-superoxide dismutase (CuZn-SOD) (activation), (NADPH) oxidase (inhibition)	Human umbilical vein endothelial cells (HUVEC) and human aorta endothelial cells (HAEC)	(259)
	Thiazolidinediones	SOD (PPRE has a Cu/Zn-SOD promoter), free fatty acid (activation)	Peripheral blood mononuclear cells	(260)
	Thiazolidinediones	Modulation of cytotrophoblast invasion, SOD, HO heme oxygenase-1 (HO-1) (activation)	Uterine tissue	(261)

### Cell Cycle Arrest

Peroxisome proliferator-activated receptor gamma agonists can induce G2/M cell cycle arrest through the stimulation of p38 MAPK in carcinoma (1) and in pancreatic cancer cells (221). PPARγ overexpression helps to stimulate the expression of cyclin-dependent kinase inhibitors p27 (222–224) and p21 (221, 222). PPARγ activation stops the cytosolic β-catenin accumulation and then decreases the expression of cyclin D1 (226–230).

### Differentiation

Peroxisome proliferator-activated receptor gamma agonists can stimulate molecules involved in well-differentiated cells, such as E-cadherin, alkaline phosphatase, keratin, and carcinoembryonic antigen (CEA). This stimulation works in opposition to the non-differentiation of cells observed in cancers (219, 221, 262–264). PPARγ agonists are involved in the stimulation of terminal differentiation in cells (216–218).

### Proliferation

Several studies have observed an anti-proliferative role played by TZDs (233, 265). Moreover, the PPARγ ligand docosahexaenoic acid (DHA) has been shown to have an anti-proliferative role in lung tumor cell cultures (234). In parallel, DHA also downregulates the cell proliferation and angiogenesis processes in breast cancer (231, 236). Rosiglitazone reduces the proliferation time of liposarcoma, but troglitazone has a limited effect in prostate, colon, and breast cancers (266–268).

### Invasion and Migration

Thiazolidinediones downregulate tumor growth and the migration of tumor cells in colon cancer cells by inducing cell differentiation (264). In the same way, TZDs arrest cell cycle G1 with a decrease in E-cadherin expression (264). To date, few studies have revealed PPARγ agonists to play a positive role in the inhibition of invasion and the migration of cancer cells.

### Apoptosis

The apoptotic process is stimulated by using a TZD in gastric cancer (241). PPARγ agonists can stimulate the expression of PTEN, a PI3K/Akt pathway inhibitor (242–245, 269), BAD, and BAX (239, 270). In the same way, PPARγ agonists can downregulate Bcl-2 expression (248) and PI3K/Akt pathway activity (249, 250).

### Inflammation

Peroxisome proliferator-activated receptor gamma agonists, such as DHA and omega-3 fatty acids EPA, are known to induce anti-inflammatory activity (252, 253). Some natural and synthetic PPARγ agonists can have a chemoprotective role by targeting inflammatory agents (257, 271). PPARγ overexpression inhibits the activity of TNF-α and NFκB (257, 272) resulting in a reduction of tumor development (273). PPARγ activation seems to act on the tumor environment, especially on inflammation (19).

### Angiogenesis

Peroxisome proliferator-activated receptor gamma agonists can modulate angiogenesis *in vitro* and *in vivo* models (274). However, some paradoxical effects of PPARγ agonists have also been observed. PPARγ agonists may enhance VEGF in tumor cells (275, 276) and may have pro- or anti-angiogenic roles depending on the cell environment (277–280).

### Oxidative Stress

Superoxide dismutase (SOD) expression is regulated by PPARγ agonists, through a Cu/Zn-SOD promoter on the PPRE (260). Numerous studies have shown PPARγ to act as an antioxidant (258, 259). PPARγ acts on macrophages by reversing and uptaking the transport of cholesterol and then decreasing the oxidative stress initiation (281–283). PPARγ ligands promote antioxidant response through the stimulation of GPx3 (273), manganese SOD (MnSOD) (284), CD36 (a scavenger receptor) (285), endothelial oxide synthase (eNOS) (286), and UCP2 (mitochondrial uncoupling protein 2) (287). In parallel, PPARγ ligands can downregulate the prooxidant response by inhibiting COX2 and iNOS (288–290).

## Conclusion

**Figure 4:**
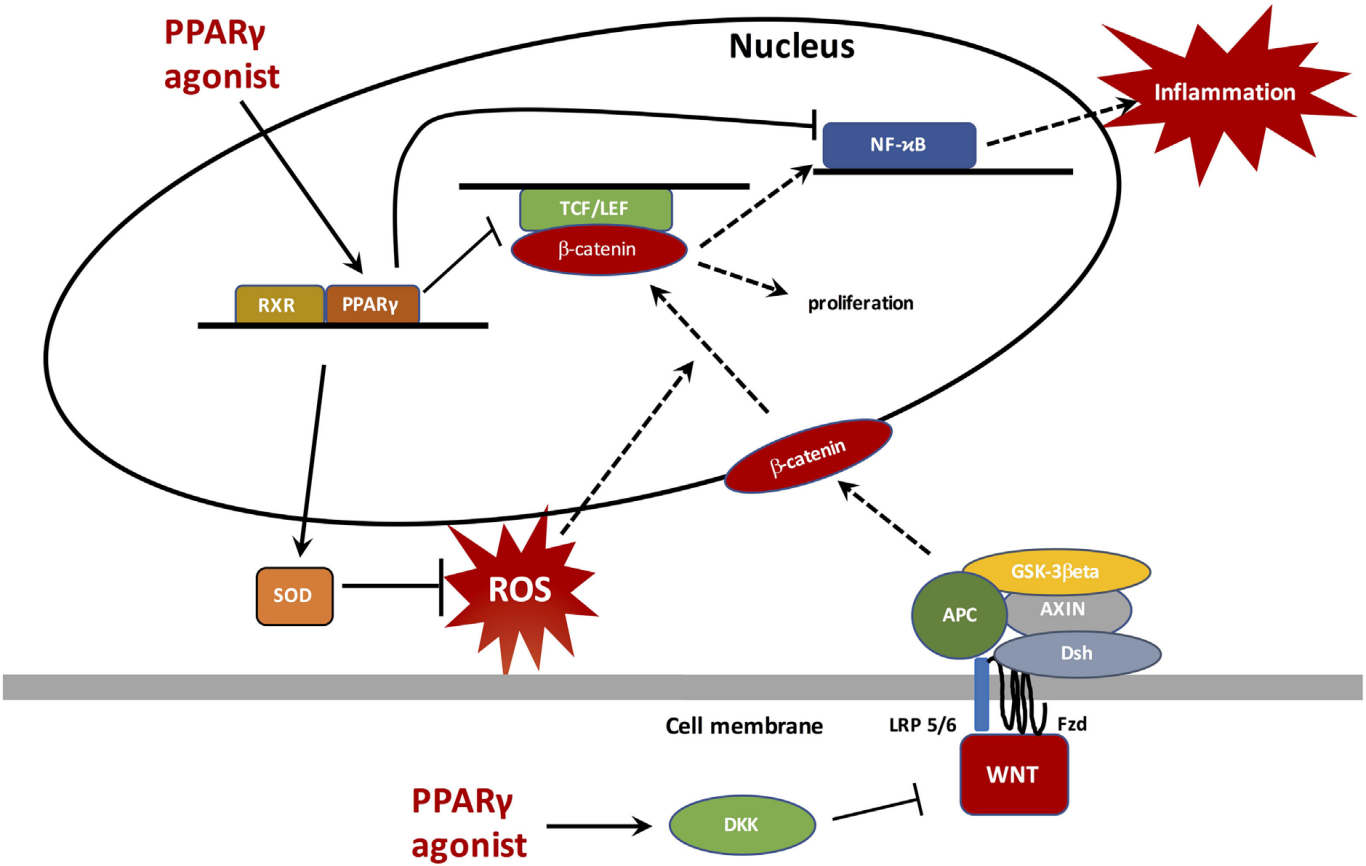
Peroxisome proliferator-activated receptor gamma (PPARγ) translocates to the nucleus to bind with retinoid X receptor (RXR) and then activates PPAR response elements (PPREs), such as superoxide dismutase (SOD) to inhibit reactive oxygen species (ROS) generation. ROS inhibition does not stimulate the β-catenin nuclear transcription and therefore does not activate proliferation processes and the NFκB pathway. PPARγ, through its anti-inflammatory role, inhibits NFκB and decreases inflammation. Through a catenin domain, PPARγ directly inhibits the TCF/LEF/β-catenin nuclear activity. In parallel, PPARγ can activate DKK, a WNT inhibitor.

Cancers are readily associated with complex inflammatory phenomena and oxidative stress that may complicate or even initiate them. In cancers, apart from certain exceptions, the canonical WNT/β-catenin signaling is generally upregulated while PPARγ is downregulated. These two major cell pathways work in an opposite manner and this partly explains their unidirectional profile observed in cancers, chronic inflammation, and oxidative stress. This results in an activation of several upstream or downstream pathways involved in carcinogenesis, such as TGF-β, NFκB, TNF-α, TGF-β, IL-6, IL-8, VEGF, iNOS, PI3K/Akt, HIF-1α, and certain target genes such as c-Myc, cyclin D1, COX2, and Bcl2. The use of PPARγ agonists in cancers could reduce both ROS production and chronic inflammation leading to a decrease in the WNT/β-catenin pathway and then an inhibition of carcinogenesis processes (Figure 4). Because of the considerable impact of cancers and inflammatory processes on mortality and morbidity rates worldwide, it is imperative to continue to find new therapeutic pathways by seeking, directly or indirectly, to inhibit the canonical system WN/β-catenin and to activate PPARγ by new agonists free of deleterious effects.

## Author Contributions

All listed authors have made substantial, direct, intellectual contributions to the study and have given their approval for its submission for publication.

## Conflict of Interest Statement

The authors declare that the research was conducted in the absence of any commercial or financial relationship that could be construed as a potential conflict of interest.

## Abbreviations

APC, adenomatous polyposis coli; CK1, casein kinase 1; COX-2, cyclooxygenase-2; EMT, epithelial-mesenchymal transition; FZD, frizzled; GSK-3β, glycogen synthase kinase-3β; LRP 5/6, low-density lipoprotein receptor-related protein 5/6; NFκB, nuclear factor-kappaB; NOX, NADPH oxidase; PPARγ, peroxisome proliferator-activated receptor gamma; PI3K-Akt, phosphatidylinositol 3-kinase-protein kinase B; ROS, reactive oxygen species; SOD, superoxide dismutase; TCF/LEF, T-cell factor/lymphoid enhancer factor; TNF- α, tumor necrosis factor alpha; TZD, thiazolidinedione.
